# Network analysis of Alcohol, Smoking and Substance Involvement Screening Test (ASSIST) 3.1 items for non-medical use of alcohol, tobacco, cannabis, prescription sedatives, prescription stimulants, and prescription opioids

**DOI:** 10.3389/fpsyt.2025.1541628

**Published:** 2025-05-16

**Authors:** Dvora Shmulewitz, Maor Daniel Levitin, Roi Eliashar, Mario Mikulincer, Shaul Lev-Ran

**Affiliations:** ^1^ Department of Psychology, Israel Center for Addiction and Mental Health, The Hebrew University of Jerusalem, Jerusalem, Israel; ^2^ Research Department, Israel Center on Addiction, Netanya, Israel; ^3^ Department of Psychology, Tel Aviv University, Tel Aviv, Israel; ^4^ Lev Hasharon Medical Center, Netanya, Israel; ^5^ Faculty of Medical and Health Sciences, Tel Aviv University, Tel Aviv, Israel

**Keywords:** network analysis, risky substance use, ASSIST 3.1, alcohol, tobacco, cannabis, prescription medications

## Abstract

**Background:**

At-risk substance use is a leading cause of preventable morbidity and mortality worldwide. The Alcohol, Smoking and Substance Involvement Screening Test 3.1 (ASSIST) is widely used to screen for such use.

**Objectives:**

Using network analysis to reframe risky substance use as a web of interacting ASSIST symptoms to provide important suggestions about potential mechanisms underlying risky use.

**Methods:**

Cross-sectional data on the ASSIST was collected via an online survey from a general population sample of Jewish adults in Israel (N=4,002; 50.4% women). Network analysis was carried out for ASSIST symptoms for non-medical use of alcohol, tobacco, cannabis, prescription sedatives, prescription stimulants, and prescription opioids. First, networks were modeled for each substance, to explore the following research questions: which symptoms were most strongly related? and what are the key symptoms that compose the networks? Second, networks were compared to determine if symptom relationships differed between substances.

**Results:**

Basic similarities were observed across substances, e.g., strongest direct associations between frequency of use and craving, and frequency of substance related problems and role interference. Role interference and craving appeared to play important roles in the networks. Differences were observed between substances in strength of associations between symptoms.

**Conclusion:**

Network structures were similar across substances, suggesting that similar intervention approaches may be appropriate, with substance-specific strategies as warranted. Among those who use substances, addressing the effects of role interference and craving in risky substance use may help reduce substance-related harms and limit progression to full blown disorder.

## 1 Introduction

Worldwide, substance use and disorders (SUD) are leading causes of preventable morbidity and mortality, and are associated with serious health, social, economic, and legal consequences (1–5). To mitigate consequences and limit disorder progression, a better understanding of the associations between SUD symptoms is needed (6). Identifying which symptoms are key to disorder etiology and maintenance can provide information for the design of targeted prevention and intervention strategies.

One method for exploring symptom structure of mental health disorders, including SUD, is network analysis, which represents disorder as a web of associated symptoms (6–11). This methodology differs from the psychometric perspective that views disorder as an underlying latent trait causing observable symptoms, which measure disorder and severity (12, 13). Rather, the symptoms themselves compose and maintain the disorder to differing extents, matching evidence that symptoms differ, e.g., may occur at different stages of disorder progression (10). By positing symptoms that interact as the underlying basis for disorder, network theory is consistent with Koob and Volkow’s three-stage cyclical model of addiction: (1) binge/intoxication, (2) withdrawal/negative affect, and (3) preoccupation/anticipation (craving) (14). In this model, substance use stimulates the brain’s reward center; reduction of the effects leads to physical and/or emotional distress, which leads to substance craving, seeking, and using again, despite negative consequences of use. Network analysis can explore how symptoms related to those stages influence each other, and suggest underlying mechanisms, such as which symptom interactions are most important for disorder development and maintenance. Within this framework, treating the disorder refers to weakening associations between symptoms that hold the network together.

Additionally, screening for early identification of and intervention for risky substance use can limit progression to full-blown disorder and associated consequences (15, 16). A widely used screen for risky substance use is the Alcohol, Smoking and Substance Involvement Screening Test 3.1 (ASSIST) (15–17). The ASSIST consists of symptoms assessing the frequency of substance use, craving, problems related to use, role interference, failure to cut down/stop use, and concern about use. Applying network models to substance-specific data can identify which ASSIST symptoms or connections between symptoms are the strongest or most important for composing the networks, and determine similarities and differences across substances. Previous studies have applied network analysis to ASSIST total scores (18) but not substance-specific symptoms, although there are network analysis studies of SUD symptoms. Some SUD symptoms, e.g., craving, problems due to use, and role interference, are similar to ASSIST symptoms, but others, e.g., withdrawal and tolerance, are not assessed. Across seven studies of alcohol use disorder symptoms, networks were dense (many symptoms were connected to each other), and consuming larger/longer than intended and health problems due to use were central, i.e., strongly connected to other symptoms (19). Studies that included specific networks for a range of substances (6, 10, 11) showed that there were similarities across substances, e.g., networks were generally dense, and using larger/longer than intended (10, 11) and craving (6) were central. There were also substance-specific differences, e.g., in terms of which symptoms were most strongly associated. Since network analysis results differ based on which symptoms are included (9, 18), it is unknown whether similar results are expected for ASSIST symptoms.

Therefore, in a general population sample from Israel, we conducted network analysis for non-medical use of common substances (alcohol, tobacco, cannabis, and prescription sedatives, stimulants, and opioid painkillers). Using a general population sample is appropriate for the ASSIST, which is designed as a population-wide screening tool and provides information across the full range of severity, from those with no or low levels of symptoms to those with high levels. Identifying specific symptoms that are more indicative of higher likelihood of risky use can inform interventions to mitigate risks of progression to more severe disorder. First, we modeled networks for each substance to explore symptoms or symptom interactions that appear most or least important to composing the networks. Second, we compared networks to determine if symptom interactions differed between substances.

## 2 Materials and methods

### 2.1 Sample

Cross-sectional data were collected in December 2023 from a general population sample of adults in Israel, similar to an epidemiological survey from 2022 (20). Respondents were recruited from a diverse panel of individuals who choose to participate in digital surveys (21). Respondents were Hebrew speaking and Jewish, since substantial adaptations would be required to include different cultural groups (22), and aged 18-70, as older individuals are less likely to participate in online surveys. To construct a quasi-representative sample of the adult, Jewish, Hebrew-speaking population in Israel, a stratified sample was drawn from the panel, utilizing specified quotas (23) based on age, gender, residential area, and religiosity. Quotas were based on Israel Census Bureau data for 2023 (24); deviations of up to 3% were allowed. Potential participants were selected in two ways within strata: all respondents surveyed in 2022 were invited to participate, as were a random sample of those who had not participated previously. Individuals who agreed to participate were screened against the quotas until the target numbers were met. Identifying information was not available to the researchers, and iPanel did not have access to survey responses, maintaining confidentiality. Survey methodology was consistent with the ICC/ESOMAR International Code on Market and Social Research (21). Procedures were performed in compliance with relevant laws and institutional guidelines and have been approved by the Institutional Review Board of the Reichman University (Approval number P_2023185; approved November 23, 2023). All participants provided electronic informed consent.

The online survey was conducted via Qualtrics (25) and assessed sociodemographics, substance use, addictive behaviors, and physical and mental health. Online surveys may be better for collecting sensitive information such as substance use (26). Participants received online gift cards worth 20 ILS upon survey completion. Quality assurance was maintained by: inviting registered individuals; 4 attention checks; and removing incomplete surveys. Of those invited (17,267), 6,765 agreed, 1,318 were excluded due to quotas, and 1,445 did not complete the survey (807 dropped out, 638 failed attention checks), for an analytical sample of 4,002.

### 2.2 Measures

The ASSIST 3.1 was administered to assess risky substance use (27). The ASSIST is a valid instrument (15), and was shown to be reliable in self-report online form (28, 29). Respondents selected substances they ever used non-medically (tobacco, alcohol, cannabis, prescription sedatives, prescription stimulants, prescription opioids, and others). For each substance ever used, respondents reported on past 3 months (1) frequency of use and (2) craving. Those with current use reported on past 3 months frequency of (3) problems due to use (problems) and (4) role interference due to use (interference). Frequency response options included: never, once or twice, 1–3 times per month, 1–4 times per week, and 5–7 times per week. Respondents were then asked (5) if others expressed concern about their use (concern) and (6) if they failed to cut down/quit use (control), and responses options were: no; yes, within the past 3 month; and yes, prior to the past 3 months. Responses to each item were weighted (27) (**Supplementary Table 1**). Two adaptations to the standard ASSIST were made: craving was assessed among those with lifetime use, not only those with current use, since craving can be experienced without use; and inclusion of “interference” for tobacco, for consistency across substances. Symptoms that were not assessed due to logical skips, e.g., problems and interference for those without current use, were coded as 0 (never) (8, 10), since definitionally they could not experience the symptom. No data were missing.

Sociodemographic variables included age, gender, religiosity, and residential area.

### 2.3 Network analysis

Network analysis comprises statistical tools used to explore relationships among symptoms, by configuring a network with nodes (observed symptoms) connected through edges (association between the symptoms). The methodology used in this exploratory study of cross-sectional data is based on recently developed standards (9, 30–33).

We used a pairwise Markov random field network model, with edges indicating the strength of conditional association between the two symptoms, controlling for all other symptoms in the model (partial correlations) (31). Specifically, we used the gaussian graphical model (ggm), which is appropriate for continuous data. Likert items with 5 responses can be considered continuous, and ggm models are robust for ordered categorical data (33–35). Since the primary research goals involve exploring overall network structures, we preferred the sparsest models, for easier visualization and interpretation. Therefore, we used regularization to estimate edge-weights (partial correlations between symptoms) while penalizing model fit for increased model complexity (i.e., including more edges) (33). The graphical least absolute shrinkage and selection operator (GLASSO) was used, which estimates some edge-weights at zero (i.e., exclusion from the network), with model fit estimated using the extended Bayesian Information Criterion (EBIC). The best-fitting model was chosen by generating 100 models with different degrees of sparsity, determined by the tuning parameter λ, which sets the penalty for increased complexity. We choose the λ that maximized model fit (lowest EBIC), using a hyperparameter γ (set to 0.5) to balance the trade-off between including false-positive edges and excluding true edges. For visualization, the matrix of edge-weights was used to graph the network, using the Fruchterman-Reingold algorithm. Blue edges indicate positive correlation and red edges indicate negative correlation, with edge thickness indicating association strength.

#### 2.3.1 Network characteristics

Network structure indicates which symptoms are connected, with density measuring the percent of present edges/total number of possible edges. Edge-weights indicate the strength of association between each pair of symptoms. Centrality measures assess how well-connected each symptom is in the network, by assessing direct connectivity, based on immediate symptom connections. *Strength* sums the absolute values of the edge-weights, while *expected influence* sums the edge-weights, to show to what extent changing one symptom would be expected to change associated symptoms.

Since the models were estimated from observed data, stability of the network characteristics was determined prior to interpretation (32).

##### 2.3.1.1 Bayesian analysis for edge stability

We conducted Bayesian analysis as sensitivity analysis to check for stability of edge inclusion (presence) or exclusion (absence) and precision of partial correlation estimates (36). For each edge, an inclusion Bayes Factor (BF) was calculated, indicating how much more likely data were using a network structure with that edge included versus a structure without that edge. BF ≥3 but <10 was considered moderate evidence for inclusion; ≥10 was considered strong evidence for inclusion; ≤1/3 but >1/10 moderate evidence for exclusion; ≤1/10 strong evidence for exclusion; and <3 and >1/3 considered “inconclusive” (37). To address uncertainty of correlation estimates, we constructed the 95% highest posterior density interval (HDI), indicating the shortest interval covering 95% of the estimate distribution. Analysis was carried out with the *easybgm* R package (38).

##### 2.3.1.2 Bootstrapping for centrality stability

Stability of centrality measures was assessed using case-drop bootstrapping, which evaluates the correlation of measures from the original sample with measures from subsamples created by iteratively “dropping” increasing percents of the sample. The information is summarized in the correlation stability coefficient (CS), which represents the maximum proportion of cases that can be dropped such that in 95% of the bootstrapped samples, the correlation is 0.7 or higher. Measures with CS values above 0.25 are considered interpretable (39). Measures with lower CS values may indicate lower stability or that all symptoms are equivalent for that measure.

##### 2.3.1.3 Bootstrapping for differences

To assess symptoms’ relative importance to the network, we determined if edge-weights for symptom pairs or centrality measures for symptoms differed. One thousand bootstrapped samples were created with resampling, and bootstrapped confidence intervals (BCI) for the difference of the two estimates were generated. Estimates were significantly different where the BCI did not include zero (32).

#### 2.3.2 Substance-specific networks

We analyzed separate networks for each substance, with three aims: (1) to identify pairs of symptoms that appear most or least important to the network, based on the relative association strength; (2) identify symptoms that may play a strong or weak role in composing the network, based on relative centrality; and (3) describe cross-substance similarities and differences in the findings from aims 1-2. Analysis was done in R, using *bootnet* (39, 40) with the *EBICglasso* function, calling *qgraph* (41, 42). To allow visual comparison of the substance-specific graphs, we used a layout averaged across all substances, with the same maximum edge weight. In each graph, the symptoms were in the same location and the same edge thickness indicated the same strength of association.

For each substance, we conducted analysis among those who ever used (alcohol=2,959; tobacco=1,992; cannabis=988; prescription sedatives=730; prescription stimulants=466; prescription opioids=400), since the ASSIST is designed for those with lifetime use. Yet, some ASSIST symptoms are only relevant to those with current use, and the ASSIST is most informative for those with current use, so we conducted supplementary sensitivity analysis among those with current use (alcohol=2,692; tobacco=1,211; cannabis=480; prescription sedatives=467; prescription stimulants=218; prescription opioids=213).

Lastly, in supplementary sensitivity analysis, we also analyzed networks in the whole dataset (N=4,002), to explore if edge-weights were similar to results among those who ever used, since in cross-substance comparisons, networks were constructed in the whole dataset to limit complications due to different samples for different substances.

#### 2.3.3 Cross-substance comparisons

For each pair of substances, we formally compared networks to see if edge-weights between the same two symptoms differed between substances (43). We calculated the Maximum (M) statistic, an omnibus test which indicates if at least one edge weight differed between two substance-specific networks. The M statistic was computed by calculating the difference in each edge-weight between the two networks and choosing the maximum difference. To determine if the M statistic was significant, 1,000 permutations, with repeated random re-assignment of group (substance) were carried out, to generate an empirical distribution for the statistic. Where significant, which edge weight(s) differ was reported. Analysis was done in R, using *NetworkComparisonTest* (44).

## 3 Results

### 3.1 Descriptives

About half the sample were women, secular; and about 40% were aged 18-34, lived in the Tel Aviv/Central region (**Table 1**). Lifetime non-medical substance use ranged from 74% (alcohol) to 10% (prescription opioids).

**Table 1:** Sample descriptives (N=4,002).

Sociodemographics			n	Percent (%)		
Gender						
Men			1981	49.5		
Women			2017	50.4		
Other			4	0.1		
Age						
18-25			757	18.9		
26-34			746	18.6		
35-49			1240	31.0		
50-70			1259	31.5		
Religiosity						
Secular			1742	43.5		
Traditional			1268	31.7		
National Religious			486	12.1		
Ultra-Orthodox			506	12.6		
Area						
Jerusalem Area			416	10.4		
Tel Aviv / Center area			2029	50.7		
Haifa / North			830	20.7		
South			571	14.3		
Judea & Samaria			156	3.9		

Non-medical substance use						
	*Lifetime use*			*Current use*		
	n		%	n		%
Tobacco	1,992		49.8	1,211		30.3
Alcohol	2,959		73.9	2,692		67.3
Cannabis	988		24.7	480		12.0
Prescription sedatives	730		18.2	467		11.7
Prescription stimulants	466		11.6	218		5.5
Prescription opioid painkillers	400		10.0	213		5.3
Other drugs^a^	173		4.3	65		1.6

^a^cocaine, amphetamines, hallucinogens, inhalants, street opioids, other drugs.

### 3.2 Substance-specific networks

**Figure 1:**
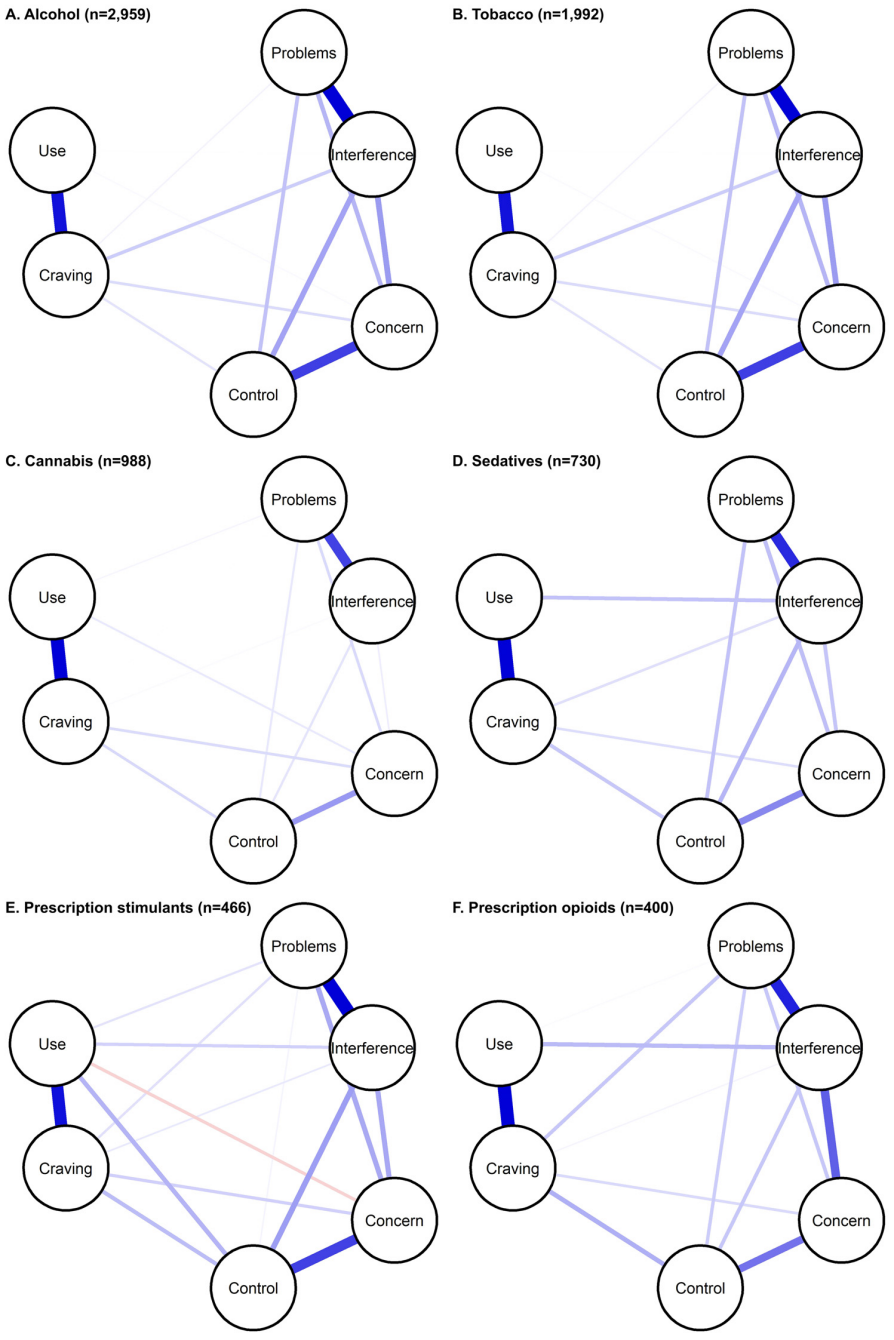
Substance-specific networks of ASSIST 3.1 symptoms, among those with lifetime use. Symptoms are shown as nodes (circles), with edges (lines) connecting symptoms that show partial correlation. Edge thickness/darkness indicates the magnitude of correlation.

Within each substance, symptoms were related to each other; covariance matrices are available as **Supplementary Material** (https://osf.io/pjbmc/). Networks for each substance among those with lifetime use (**Figure 1**) and current use (**Supplementary Figure 1**) are shown.

#### 3.2.1 Network characteristics

Across substances, networks were dense (73%-100%), with similar average edge weights (**Supplementary Table 2**). Results were similar among those with current use, and edge-weights among those with lifetime and current use were highly correlated (0.900-0.997) (**Supplementary Table 2**). In the Bayesian sensitivity analysis, evidence of edge presence or absence was conclusive for the following number of edges: tobacco (14); alcohol (15 [all]); cannabis (13); prescription sedatives (12); prescription stimulants (12); and prescription opioids (11). The same edges were generally included in main (regularization) and Bayesian analysis (**Supplementary Table 3**). Where Bayesian analysis excluded edges found in main analysis for all substances except cannabis), those generally showed low correlation. Additionally, some edges with low correlation estimates had 95% HDI overlapping with 0. These results suggest that edges with correlations on the lower range may be less reliable. Edge weights were generally positive and ranged as follows: alcohol: 0-0.48; tobacco: 0-0.78; cannabis: 0-0.61; prescription sedatives: -0.09-0.48; prescription stimulants: 0-0.51; prescription opioids: 0-0.52 (**Supplementary Table 4**). Results were similar from sensitivity analysis among those with current use. Furthermore, results from sensitivity analysis in the whole sample were similar (**Supplementary Figure 2**, **Supplementary Table 5**), with stable edge-weights (**Supplementary Table 6**) and high correlation between edge weights among those with lifetime use and the whole sample (0.972-0.998).

Across substances, the strongest correlations were observed between frequency of use and craving, and problems and interference due to use. These correlations were significantly greater than the correlations observed for almost all other symptom pairs (**Supplementary Figure 3**). The third strongest correlations observed were concern and control for alcohol, tobacco, cannabis and prescription sedatives, and interference and concern for prescription stimulants and prescription opioids. Those pairs also showed the strongest associations among those with current use (**Supplementary Table 4**) and in the whole sample (**Supplementary Table 5**). Across substances, among those with current use, frequency of use generally had low direct correlations with other symptoms (besides craving). Last, many symptom pairs showed different strength of association across substances.

#### 3.2.2 Centrality measures

**Figure 2:**
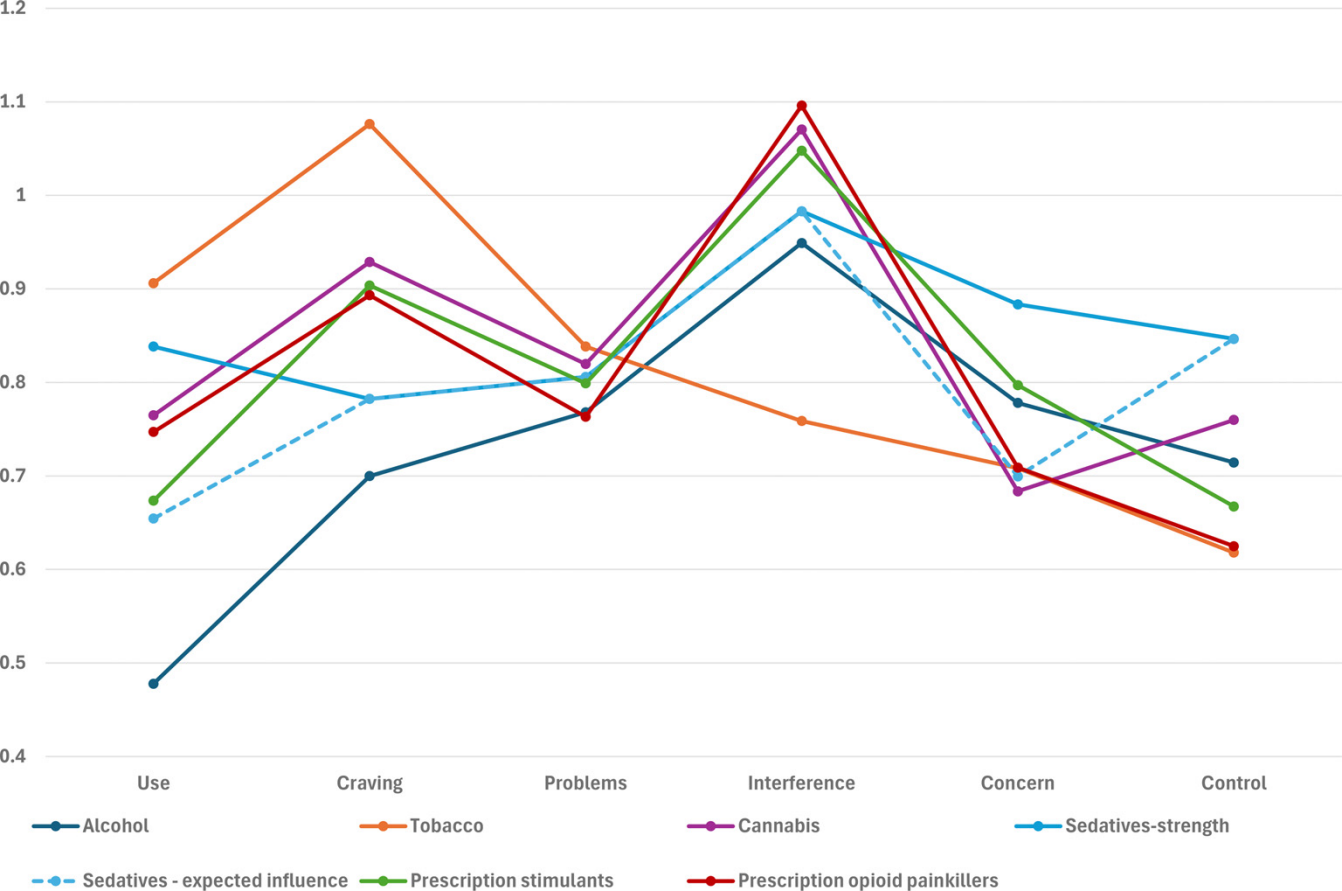
Substance-specific centrality measures, among those with lifetime use. Each series represents both strength and expected influence centrality as they were the same, except for sedatives.

Stability of centrality measures is shown in **Supplementary Table 7** and **Supplementary Figure 4**. For most substances, measures showed adequate stability and can be used to suggest symptoms with stronger or weaker connectivity, except for prescription sedatives, which had lower stability for strength. Across substances (**Figure 2**), frequency of role interference showed strongest connections with other symptoms, except for tobacco (**Supplementary Figure 5**). Frequency of craving showed the highest connectivity for tobacco, and was among the highest for cannabis and prescription stimulants and opioids, but showed low connectivity for alcohol. Similar results were observed among those with current use (**Supplementary Figure 6**). Low connectivity was also observed for frequency of use among those with current use for all substances except tobacco.

#### 3.2.3 Statistical comparisons

Significant differences in edge weights were observed for alcohol and tobacco and each other substance, except alcohol and prescription sedatives (**Table 2**). Specifically, the edge weights for frequency of use and three symptoms (craving; interference; and concern) differed for tobacco and all other substances and for alcohol and most other substances. For example, the edge weight for use and craving was highest for tobacco. Other edges that differed in the majority of comparisons included frequency of interference and concern, with tobacco showing the lowest edge weight (**Supplementary Table 8**).

**Table 2:** Comparing edge-weights across substance-specific networks.

Substance	Tobacco	Cannabis	Prescription Sedatives	Prescription stimulants	Prescription opioids
Alcohol	**M=0.217, p=.001**	**M=0.150, p=.043**	M=0.137, p=.081	**M=0.172, p=.022**	**M=0.153, p=.048**
Tobacco	**--**	**M=0.150, p=.008**	**M=0.197, p=.001**	**M=0.273, p=.001**	**M=0.266, p=.001**
Cannabis		--	M=0.160, p=.140	M=0.198, p=.103	M=0.191, p=.123
Prescription Sedatives			--	M=0.167, p=.265	M=0.191, p=.165
Prescription stimulants				--	M=0.145, p=.729

M statistics (omnibus test for differences in edge-weights) are shown. Networks that differ significantly are shown in bold; the symptom pairs with significantly different edge-weights are shown in **Supplementary Table 8**.

## 4 Discussion

Network analysis of the ASSIST 3.1 screen for risky non-medical use of common substances (alcohol, tobacco, cannabis, and prescription sedatives, stimulants, and opioid painkillers) in a general population sample of Jewish adults in Israel provided insights into interactions between symptoms. Across the substance-specific networks, the same symptom pairs showed strong associations: frequency of current use and craving, and problems and role interference due to use. Frequency of role interference showed strong centrality for all substances except tobacco, which showed strong centrality for craving. Low centrality was observed for frequency of use except for tobacco. Alcohol and tobacco showed differences in association strength between specific symptom pairs with other substances. These results add information about the relationships between symptoms and similarities and differences across substances, and suggest both overall and substance-specific symptoms/symptoms pairs that may be highly active in the progression of risky use.

Some symptom pairs showed similar association across substances. Frequency of use and frequency of craving was among the most strongly correlated pairs, consistent with numerous studies showing association of substance use and craving (45, 46). Yet, association strength differed between substances, e.g., was weaker for alcohol and stronger for tobacco, suggesting that this connection may be differentially important across substances. For example, changing craving may have a greater effect on use for tobacco than for other substances. For all substances, frequency of use showed mostly weak direct correlations with other symptoms (besides craving). This suggests that once the network is activated by substance use, frequency of use is not as directly important as other symptoms in determining extent of problems. This is consistent with the Diagnostic and Statistical Manual of Mental Disorders (DSM) framework, which does not include frequency of use as an SUD symptom (13). Thus, the binge/intoxication stage of Koob and Volkow’s addiction cycle (14) could be indicated by impaired control over use (which was related to other symptoms in our networks) rather than frequency of use. Additionally, among those with current use, frequency of use showed low centrality for most substances, again suggesting less direct influence on the other symptoms. Rather, frequency of use appears to be associated with other symptoms indirectly through craving, suggesting that treating craving may reduce the risks of progressing from use to disorder.

Moreover, craving showed strong centrality for tobacco, and also cannabis and prescription opioids, similar to network analysis of DSM-5 SUD criteria (6), consistent with psychometric studies showing the importance of craving as an SUD symptom (47–49). Craving may also be a proxy for the DSM-5 criterion of using more/for longer than intended, which is not included in the ASSIST but was shown to be central for DSM-5 SUD criteria (10, 11, 19). Yet, craving showed lower centrality for alcohol, similar to findings that baseline craving showed weaker association with subsequent substance use for alcohol than other substances (49). Further studies of systems dynamics are needed to determine the differential role of frequency of use and craving in the transition to risky use and SUD across substances.

Another of the most strongly associated pairs across substances was frequency of problems related to use and role interference. These correlations are reasonable, and may indicate more severe problematic use that affects day-to-day functioning (50). Strategies aimed at reducing negative consequences of use may be useful for addressing such problems. Also, this coupling may be partially due to time frame, since only people with current use could endorse those experiences; but those may be most likely to need and receive intervention.

Moreover, frequency of role interference showed strong centrality across most substances, similar to a study of DSM SUD criteria (10). Endorsing a symptom with strong connections to other symptoms could indicate more severe risky use. Another possibility is that role interference may have a functional aspect within Koob and Volkow’s three-stage model (14). One, interference could reflect lack of motivation or interest in doing other activities typified by the withdrawal/negative affect stage. Two, the preoccupation/anticipation stage is characterized by impairment in executive function, possibly leading to poor choices, such as using despite its effects on other activities. Failure to fulfill responsibilities might increase distress, and craving, and then use, feeding into the addiction cycle. Further studies are warranted to more fully understand the role of this symptom.

These network analyses suggest mechanisms underlying ASSIST-defined risky substance use, with important implications across substances. The underlying similarities suggest that the same overall approach may be appropriate, with substance-specific strategies as warranted. First, some symptom pairs showed different strength of association across substances, suggesting differential influence of symptoms on each other. Thus, specific interventions should target the most strongly associated symptom pairs for each substance. Additional studies should identify the source of these differences to provide further understanding of the mechanisms underlying substance addictions in general and substance-specific nuances. Second, craving is a complex construct (45, 51, 52) and some types of craving may be more relevant to specific substances than others, which may impact how craving would be treated. Similarly, the type and extent of consequences and role interference may depend on the social context and physiological effects, which may differ by substance, and require somewhat different approaches. Furthermore, for more efficient screening of the general population in primary care to identify those likely to benefit most from intervention, a shorter version of the ASSIST, consisting of craving and role interference, should be explored. Lastly, symptom relationships may not be the same within an individual. Network analysis of ecological momentary assessment data (45, 53–55), which follows individuals over time with frequent measurements of actual behavior, may be better for developing personalized treatment strategies.

### 4.1 Limitations

First, cross-sectional analysis cannot determine the directionality of the correlations between symptoms, but network analysis as applied here is an exploratory method to provide insight into symptom interactions to develop further hypotheses. Longitudinal studies are needed to better understand network progression and dynamics, and investigate between-individual and within-individual network effects. Second, the ASSIST includes symptoms considered most relevant to screening for risky use, but other symptoms may be underlying the observed relationships. Studies including other measures of risky use and SUD can build on this study to fully understand important symptom interactions. Third, respondents were limited to those able to participate in the online survey, leading to potential selection bias, but quotas were used to collect a quasi-representative sample of the Jewish, adult, Hebrew-speaking population of Israel, with respect to key sociodemographic factors. Fourth, the sample was not representative of important sectors of the population that would need methodological adaptations, e.g., those with cultural differences or less likely to complete online surveys. More representative samples of the entire Israeli population should be collected for future studies. Nevertheless, the sample is most culturally similar to and may be most generalizable to Western populations. Fifth, only Hebrew speakers were included, but >90% of Jews in Israel have mastery of Hebrew (56). Sixth, participants responded based on their understanding of the questions, but a standard, validated screening instrument was used. There may be reluctance to report illegal or stigmatized behaviors, which should be lessened by using a confidential online platform (26). Seventh, sample sizes were different for each substance, which likely affected the stability of the network models, and no information was available about substances less prevalent in Israel. Last, future studies should explore whether networks differ by age or gender.

## 5 Conclusions

By reframing at-risk substance use as a network comprised of mutually influencing symptoms, this study suggested underlying mechanisms, with implications for potential interventions. First, across substances, role interference and craving seem to be important in directly composing the networks, while frequency of use appears more indirect. Second, despite the basic similarities between the substance networks, differences were observed, suggesting that while a similar approach may be appropriate across substances, substance-specific strategies are also warranted. Additionally, results can be leveraged to develop prevention strategies that are applicable on the general population level. While additional research is needed, these findings provide information to further progress towards mitigating the negative consequences of risky substance use.

## Data Availability

The datasets presented in this article are not readily available because of proprietary issues. Requests to access the datasets should be directed to mario.mikulincer1@mail.huji.ac.il.
