# Supplementary material for: Network analysis of Alcohol, Smoking and Substance Involvement Screening Test (ASSIST) 3.1 items for non-medical use of alcohol, tobacco, cannabis, prescription sedatives, prescription stimulants, and prescription opioids
Source: Front Psychiatry. 2025 May 16;16:1541628. doi: 10.3389/fpsyt.2025.1541628 (PMC12122760; doi:10.3389/fpsyt.2025.1541628)
Supplement: Supplementary file 1 [file DataSheet1.zip › Supplementary Table 1 and Supplementary Figures.docx]

**Supplementary Table 1: ASSIST 3.1 symptoms and scoring**

| **Symptom** | **Question** | **Response options** | **Weighted score** |
| --- | --- | --- | --- |
| Current use | In the past 3 months, how often have you used (substance)? | 1 = never | 0 |
|  |  | 2 = once or twice | 2 |
|  |  | 3 = 1-3 times a month | 3 |
|  |  | 4 = 1-4 times a week | 4 |
|  |  | 5 = 5-7 times a week | 6 |
| Craving | During the past 3 months how often have you had a strong desire or urge to use (substance)? | 1 = never | 0 |
|  |  | 2 = once or twice | 3 |
|  |  | 3 = 1-3 times a month | 4 |
|  |  | 4 = 1-4 times a week | 5 |
|  |  | 5 = 5-7 times a week | 6 |
| Problems | During the past 3 months how often has your use of (substance) led to health, social, legal or financial problems? | 1 = never | 0 |
|  |  | 2 = once or twice | 4 |
|  |  | 3 = 1-3 times a month | 5 |
|  |  | 4 = 1-4 times a week | 6 |
|  |  | 5 = 5-7 times a week | 7 |
| Interference | During the past 3 months how often have you failed to do what was normally expected of you because of your use of (substance)? | 1 = never | 0 |
|  |  | 2 = once or twice | 5 |
|  |  | 3 = 1-3 times a month | 6 |
|  |  | 4 = 1-4 times a week | 7 |
|  |  | 5 = 5-7 times a week | 8 |
| Concern | Has a friend or relative or anyone else ever expressed concern about your use of (substance)? | 1 = no | 0 |
|  |  | 2 = yes, but not in past 3 months | 3 |
|  |  | 3 = yes, in past 3 months | 6 |
| Control | Have you ever tried to cut down or stop using (substance) but failed? | 1 = no | 0 |
|  |  | 2 = yes, but not in past 3 months | 3 |
|  |  | 3 = yes, in past 3 months | 6 |

"Current use", "craving", "concern" and "control" items were asked to those who ever used the substance; "problems" and "interference" items were asked to those who used in the past 3 months (current use), and were considered "never" for those without current use.

**Supplementary Figure 1: Networks of ASSIST items, among those with past three month use**


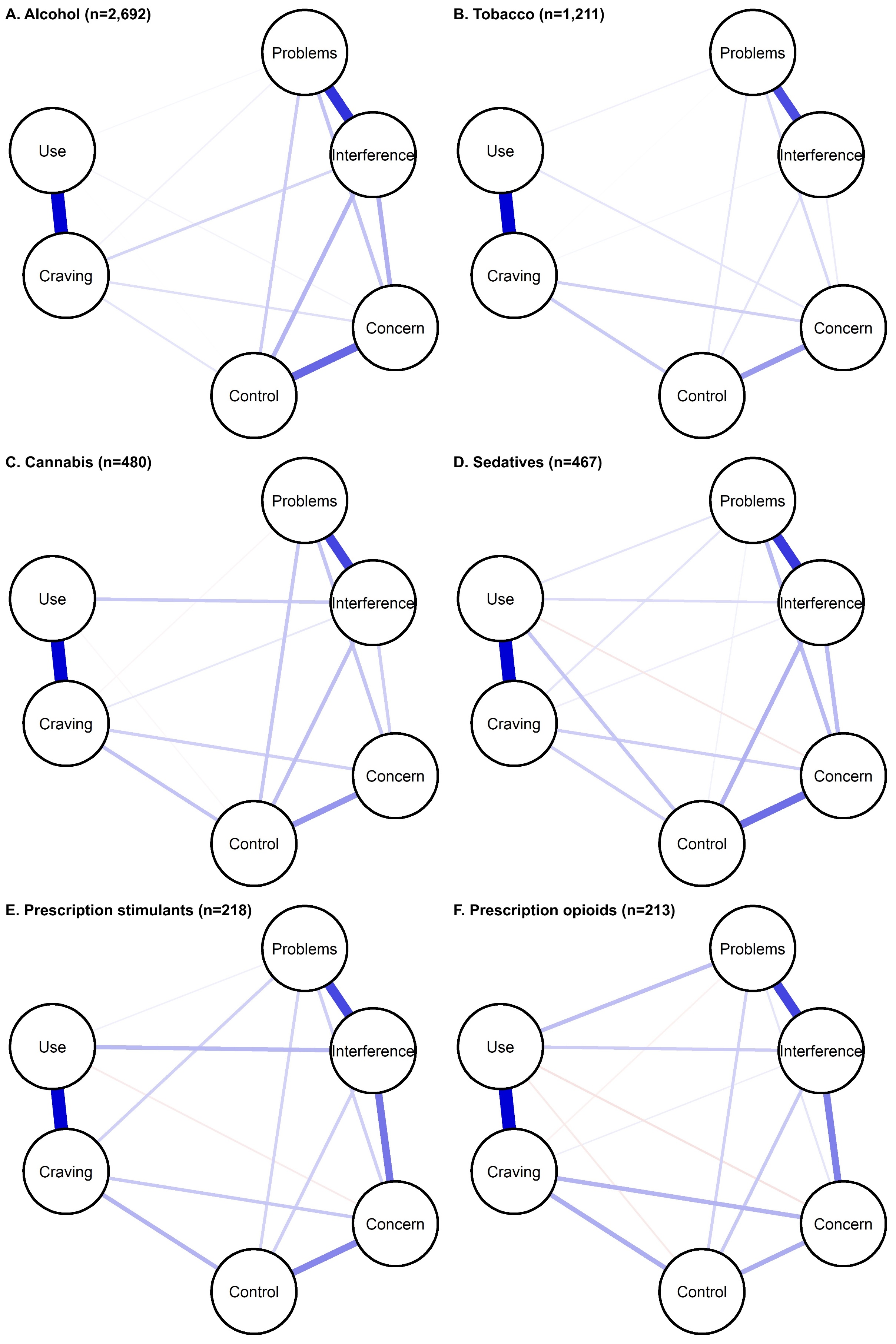


Note: Symptoms are shown as nodes (circles), with edges (lines) connecting symptoms that show partial correlation. Edge thickness/darkness indicates the magnitude of correlation.

**Supplementary Table 2. Network architecture**

| **Substance** | **Symptoms** | **Density** (number of edges/possible edges) | | **Average edge weights** | | **Correlations** between edge weights among networks from those with lifetime use and those with current use |
| --- | --- | --- | --- | --- | --- | --- |
|  |  | Lifetime use | Current use | Lifetime use | Current use |  |
| Alcohol | 6 | 14/15=93.3% | 13/15=86.7% | 0.15 | 0.14 | 0.997 |
| Tobacco | 6 | 13/15=86.7% | 12/15=80.0% | 0.16 | 0.14 | 0.990 |
| Cannabis | 6 | 11/15=73.3% | 13/15=86.7% | 0.17 | 0.16 | 0.972 |
| Prescription Sedatives | 6 | 15/15=100.0% | 12/15=80.0% | 0.16 | 0.14 | 0.913 |
| Prescription stimulants | 6 | 13/15=86.7% | 11/15=73.3% | 0.18 | 0.14 | 0.900 |
| Prescription opioids | 6 | 12/15=80.0% | 12/15=80.0% | 0.16 | 0.15 | 0.951 |

"Lifetime use" refers to those who reported non-medical use at least once in lifetime; "Current use" refers to those who reported non-medical use at least once in the past three months

**Supplementary Table 3 is found as an Excel spreadsheet.**

**Supplementary Table 4. Edge weights, substance specific networks**

|  | **Alcohol** | | **Tobacco** | | **Cannabis** | | **Sedatives** | | **Prescription stimulants** | | **Prescription opioids** | |
| --- | --- | --- | --- | --- | --- | --- | --- | --- | --- | --- | --- | --- |
| **Edges** | Lifetime | Current | Lifetime | Current | Lifetime | Current | Lifetime | Current | Lifetime | Current | Lifetime | Current |
| Use – craving | 0.44 | 0.39 | 0.78 | 0.64 | 0.61 | 0.52 | 0.45 | 0.25 | 0.51 | 0.30 | 0.52 | 0.42 |
| Use – problems | 0.00 | 0.00 | 0.04 | 0.00 | 0.00 | -0.04 | 0.06 | 0.00 | 0.02 | 0.00 | 0.13 | 0.04 |
| Use – interference | 0.01 | 0.00 | 0.00 | -0.04 | 0.15 | 0.07 | 0.09 | 0.00 | 0.14 | 0.00 | 0.10 | 0.01 |
| Use – concern | 0.02 | 0.03 | 0.08 | 0.04 | 0.00 | 0.00 | -0.09 | 0.00 | 0.00 | 0.00 | 0.00 | 0.00 |
| Use – control | 0.01 | 0.03 | 0.00 | 0.00 | 0.00 | 0.05 | 0.15 | 0.10 | 0.00 | 0.00 | 0.00 | 0.00 |
| Craving – problems | 0.03 | 0.02 | 0.01 | 0.00 | 0.00 | 0.00 | 0.06 | 0.06 | 0.12 | 0.07 | 0.00 | 0.00 |
| Craving – interference | 0.10 | 0.10 | 0.03 | 0.01 | 0.08 | 0.11 | 0.05 | 0.07 | 0.03 | 0.02 | 0.07 | 0.06 |
| Craving – concern | 0.08 | 0.08 | 0.12 | 0.15 | 0.09 | 0.07 | 0.10 | 0.04 | 0.08 | 0.12 | 0.13 | 0.15 |
| Craving – control | 0.06 | 0.06 | 0.13 | 0.12 | 0.14 | 0.13 | 0.13 | 0.15 | 0.16 | 0.15 | 0.17 | 0.18 |
| Problems – interference | 0.48 | 0.47 | 0.57 | 0.54 | 0.51 | 0.45 | 0.48 | 0.44 | 0.44 | 0.36 | 0.47 | 0.44 |
| Problems – concern | 0.14 | 0.14 | 0.13 | 0.13 | 0.16 | 0.19 | 0.17 | 0.19 | 0.12 | 0.15 | 0.06 | 0.08 |
| Problems – control | 0.12 | 0.12 | 0.08 | 0.09 | 0.15 | 0.19 | 0.03 | 0.02 | 0.11 | 0.10 | 0.10 | 0.12 |
| Interference – concern | 0.19 | 0.19 | 0.06 | 0.07 | 0.15 | 0.18 | 0.17 | 0.19 | 0.32 | 0.37 | 0.31 | 0.38 |
| Interference – control | 0.17 | 0.18 | 0.09 | 0.10 | 0.18 | 0.22 | 0.19 | 0.18 | 0.12 | 0.11 | 0.14 | 0.13 |
| Concern – control | 0.35 | 0.35 | 0.32 | 0.29 | 0.29 | 0.26 | 0.36 | 0.36 | 0.28 | 0.27 | 0.21 | 0.26 |

"Lifetime" refers to those who reported non-medical use at least once in lifetime; "Current" refers to those who reported non-medical use at least once in the past three months

**Supplementary Figure 2: Networks of ASSIST items, among the whole sample (N=4,002)**


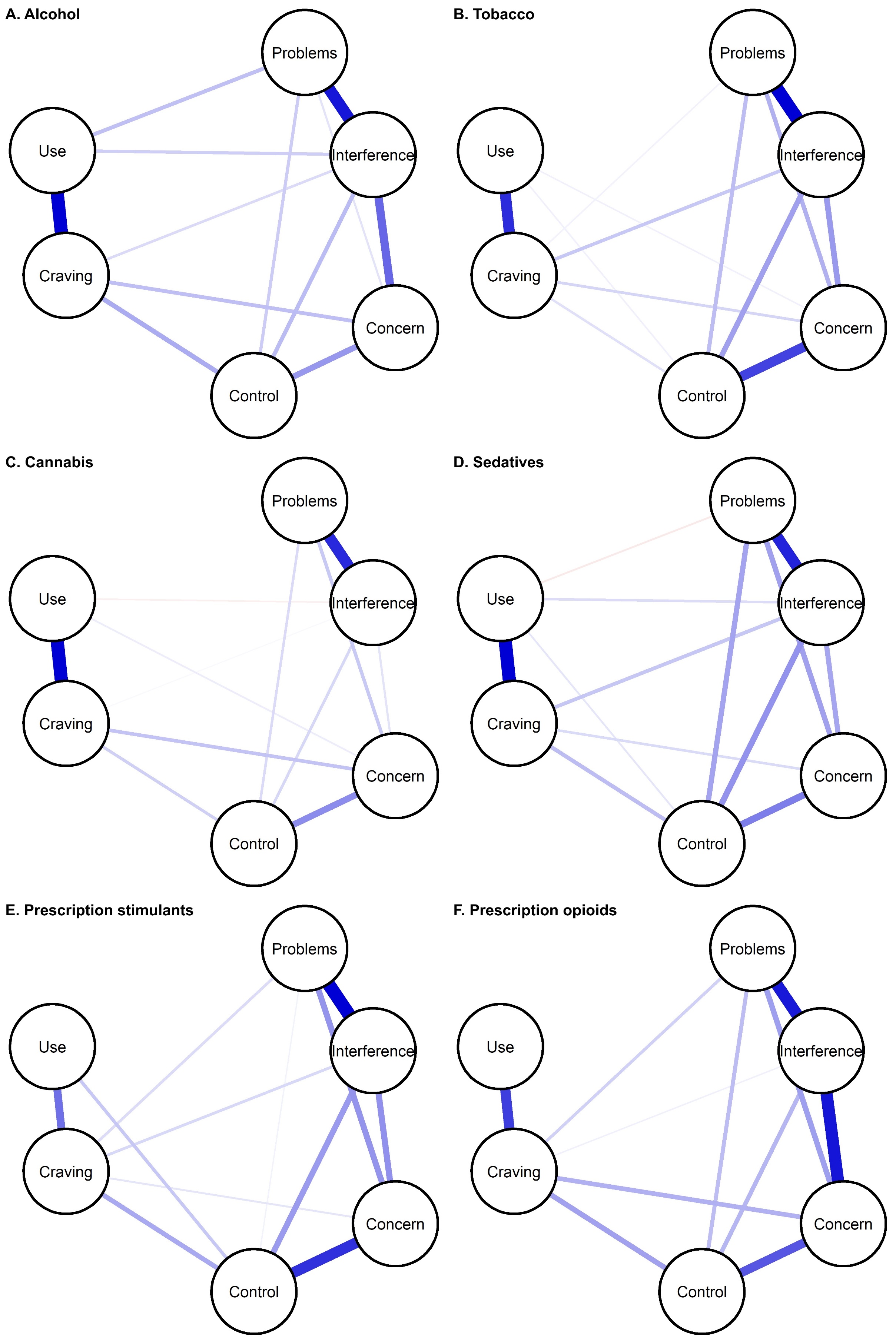


Note: Symptoms are shown as nodes (circles), with edges (lines) connecting symptoms that show partial correlation. Edge thickness/darkness indicates the magnitude of correlation.

**Supplementary Table 5. Edge weights, substance specific networks, whole sample (N=4,002)**

| **Edges** | Alcohol | Tobacco | Cannabis | Sedatives | Prescription stimulants | Prescription opioids |
| --- | --- | --- | --- | --- | --- | --- |
| Use – craving | 0.60 | 0.82 | 0.71 | 0.62 | 0.62 | 0.66 |
| Use – problems | 0.02 | 0.04 | 0.00 | 0.07 | 0.04 | 0.17 |
| Use – interference | 0.00 | 0.00 | 0.15 | 0.09 | 0.17 | 0.13 |
| Use – concern | 0.03 | 0.08 | 0.00 | -0.06 | -0.05 | -0.07 |
| Use – control | 0.00 | 0.00 | -0.02 | 0.14 | 0.00 | -0.05 |
| Craving – problems | 0.03 | 0.01 | -0.03 | 0.07 | 0.11 | -0.03 |
| Craving – interference | 0.10 | 0.03 | 0.06 | 0.04 | 0.00 | 0.04 |
| Craving – concern | 0.08 | 0.15 | 0.13 | 0.12 | 0.13 | 0.19 |
| Craving – control | 0.06 | 0.17 | 0.17 | 0.12 | 0.18 | 0.21 |
| Problems – interference | 0.49 | 0.58 | 0.52 | 0.49 | 0.45 | 0.48 |
| Problems – concern | 0.14 | 0.12 | 0.16 | 0.17 | 0.12 | 0.07 |
| Problems – control | 0.12 | 0.08 | 0.16 | 0.03 | 0.11 | 0.12 |
| Interference – concern | 0.19 | 0.06 | 0.14 | 0.17 | 0.34 | 0.33 |
| Interference – control | 0.18 | 0.09 | 0.18 | 0.19 | 0.12 | 0.14 |
| Concern – control | 0.36 | 0.33 | 0.29 | 0.35 | 0.29 | 0.22 |

**Supplementary Table 6. Network architecture, whole sample (N=4,002)**

| **Substance** | **Symptoms** | **Density** (number of edges/possible edges) | **Average edge weights** | **Correlation** between edge weights in whole sample and in those who ever used |
| --- | --- | --- | --- | --- |
| Alcohol | 6 | 14/15=93.3% | 0.16 | 0.981 |
| Tobacco | 6 | 13/15=86.7% | 0.17 | 0.998 |
| Cannabis | 6 | 13/15=86.7% | 0.18 | 0.994 |
| Prescription Sedatives | 6 | 15/15=100% | 0.17 | 0.972 |
| Prescription stimulants | 6 | 14/15=93.3% | 0.18 | 0.989 |
| Prescription opioids | 6 | 15/15=100% | 0.17 | 0.982 |

**Supplementary Table 7: Stability of centrality measures using case-dropping bootstrap correlation stability coefficients**

| **Substance** | **Strength** | | **Expected Influence** | |
| --- | --- | --- | --- | --- |
|  | Lifetime use | Current use | Lifetime use | Current use |
| Alcohol | *0.75* | *0.75* | *0.75* | *0.75* |
| Tobacco | *0.75* | *0.68* | *0.75* | *0.75* |
| Cannabis | *0.64* | *0.53* | *0.71* | *0.60* |
| Prescription sedatives | 0.05 | *0.68* | *0.53* | *0.71* |
| Prescription stimulants | *0.46* | *0.64* | *0.60* | *0.64* |
| Prescription opioids | *0.42* | *0.46* | *0.60* | *0.57* |

"Lifetime use" refers to those who reported non-medical use at least once in lifetime; "Current use" refers to those who reported non-medical use at least once in the past three months

Values >0.25 are considered interpretable and shown in italics

**Supplementary Figure 3: Differences between edge weights, substance specific networks**


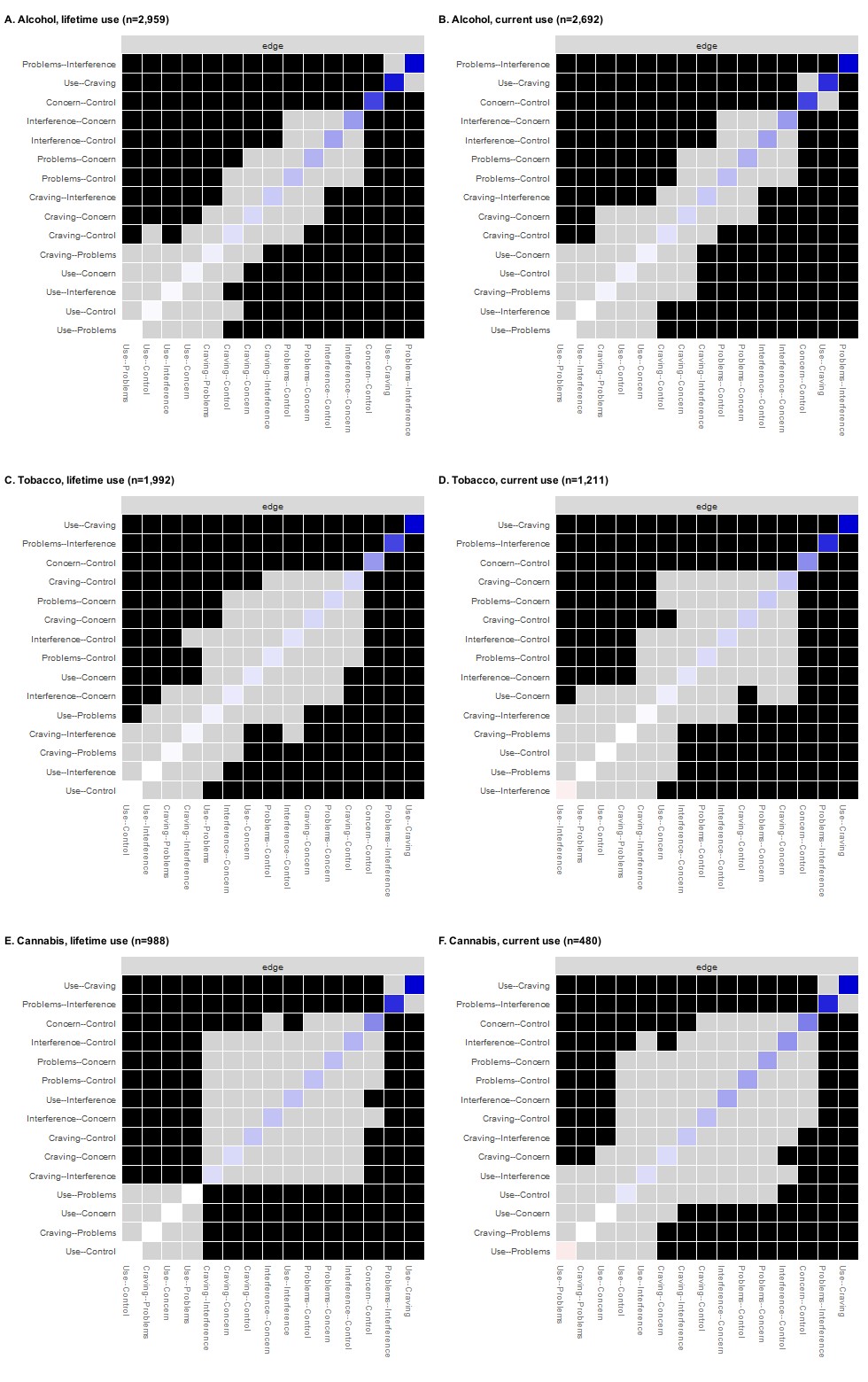


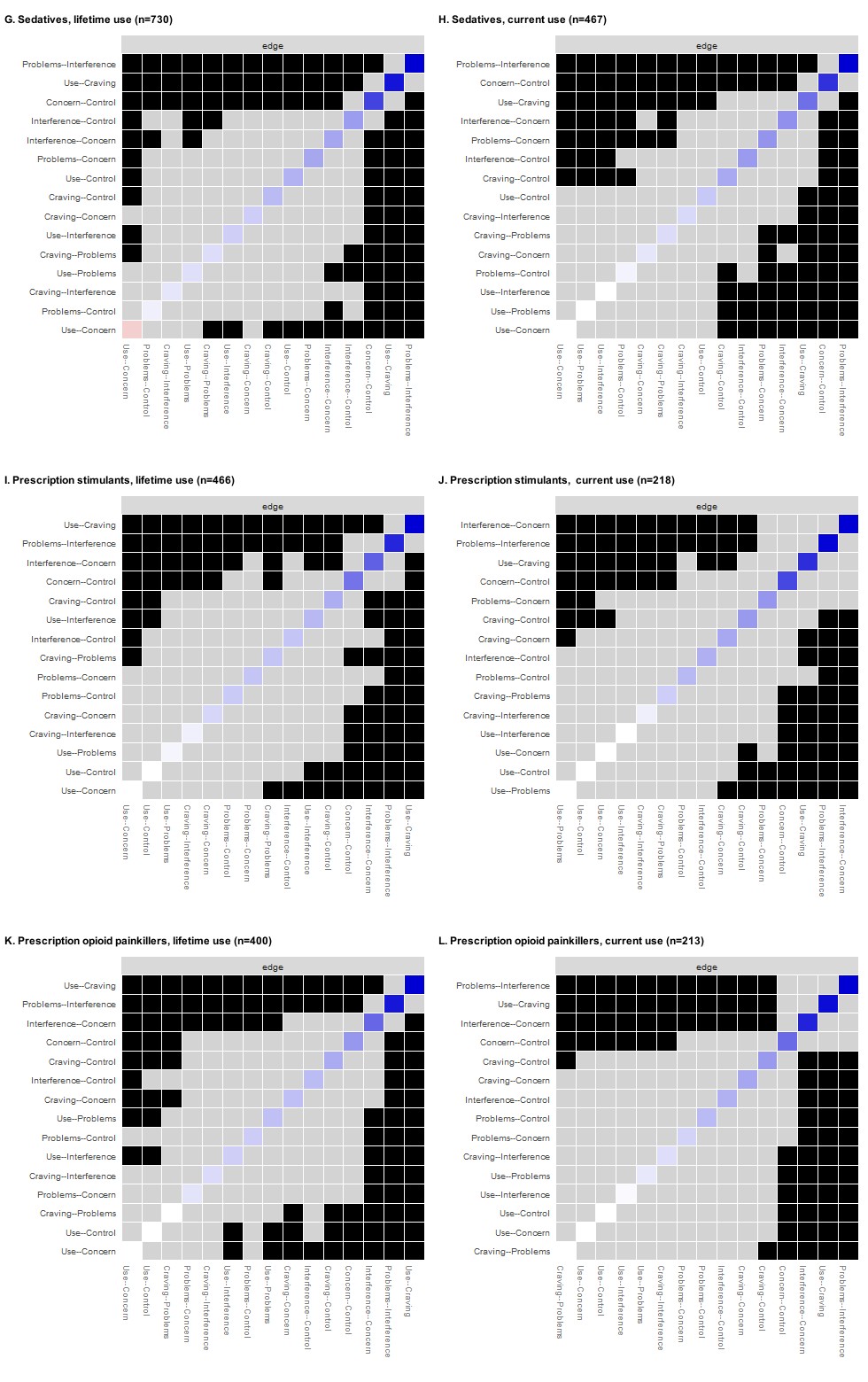


Edge weights for each pair of symptoms (partial correlation) are shown on the diagonal, with dark blue indicating high weights, and white indicating low weights. Black squares indicate edge weights that differ significantly from each other (p<.05).

**Supplementary Figure 4: Stability of centrality measures, substance specific networks**


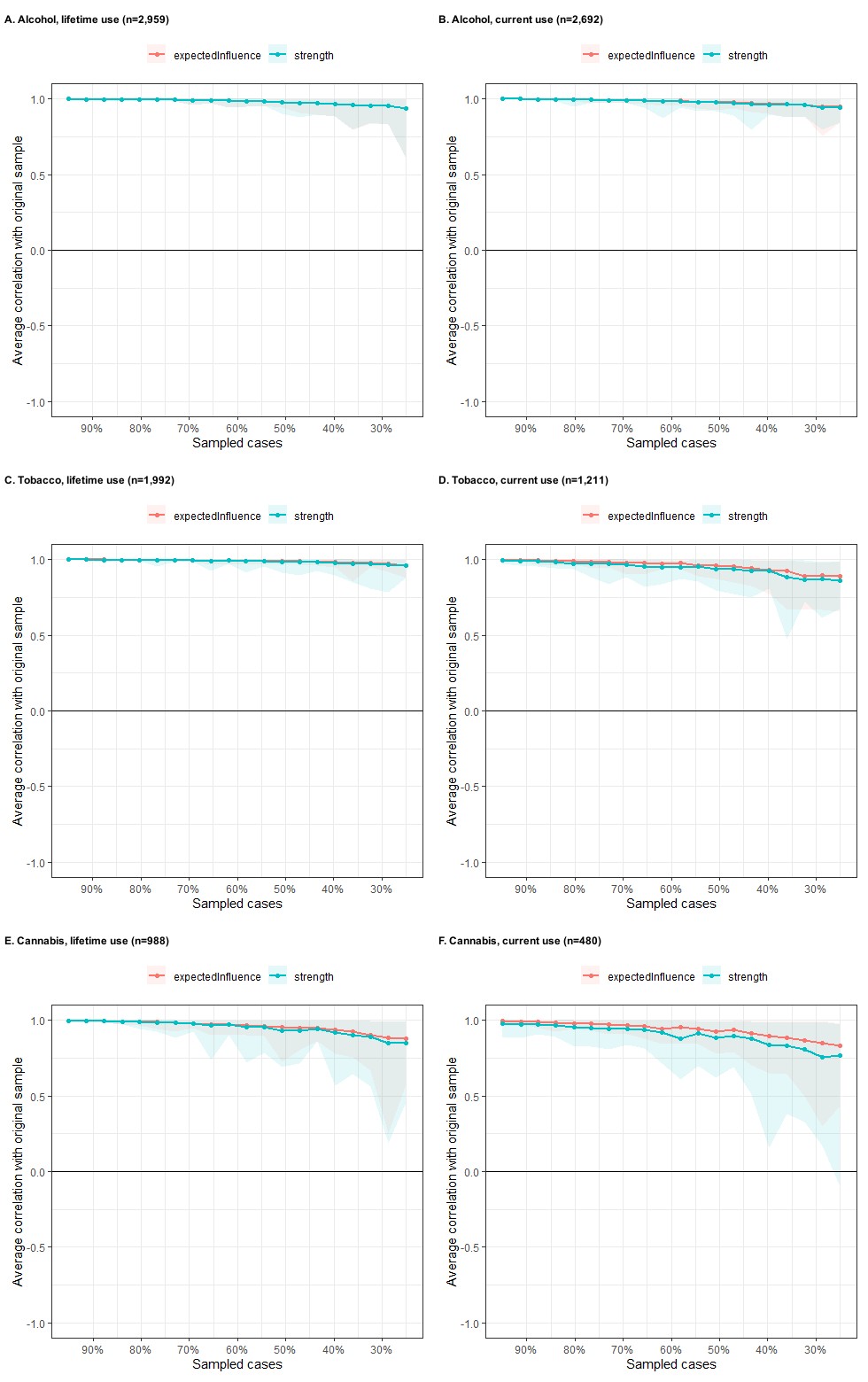


**
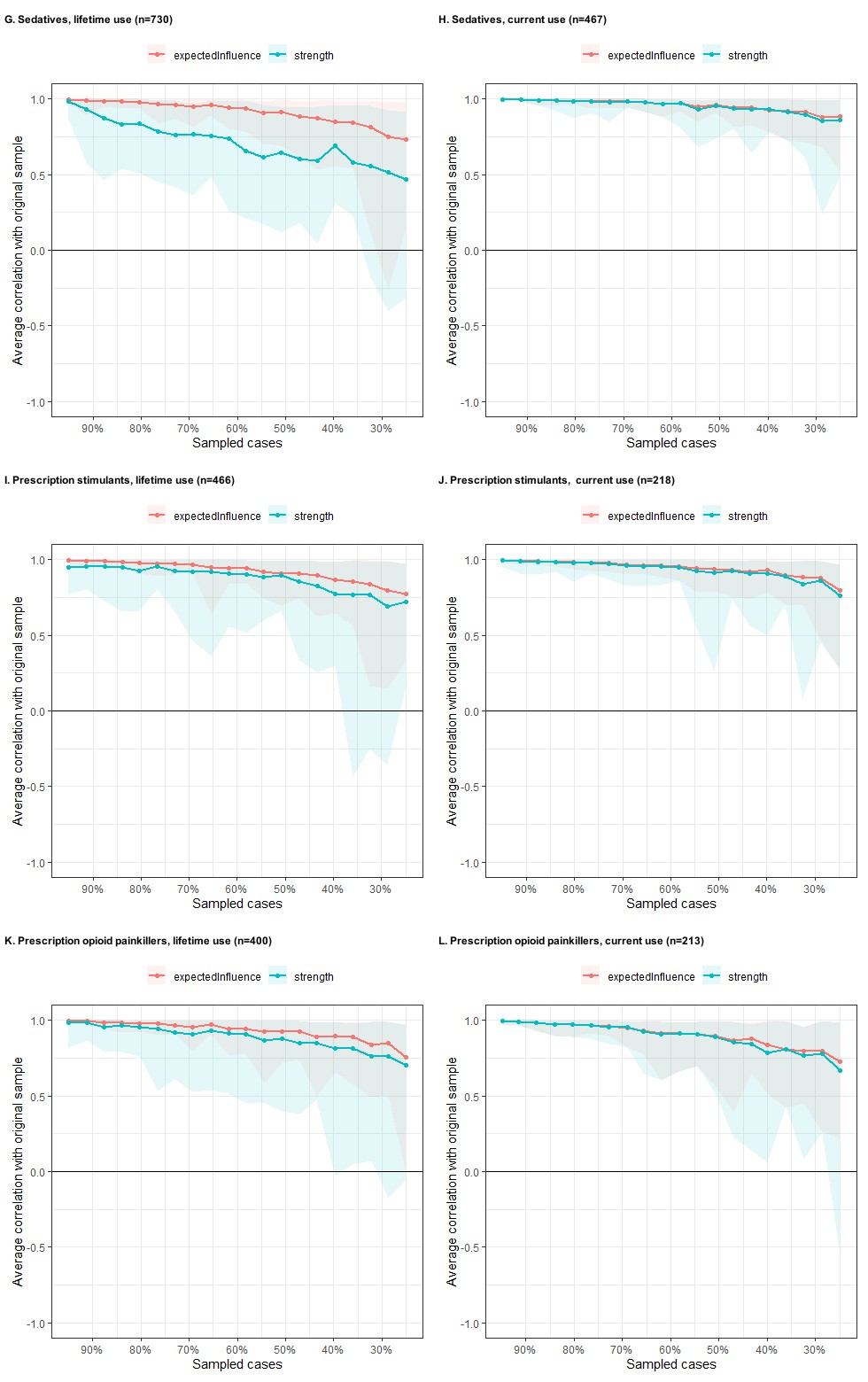
**

Graphs show the correlation between centrality measures in the original sample with centrality measures in the sub-samples, as a function of sample size; straighter lines indicate greater stability.

**Supplementary Figure 5: Difference between centrality measures, substance specific networks**


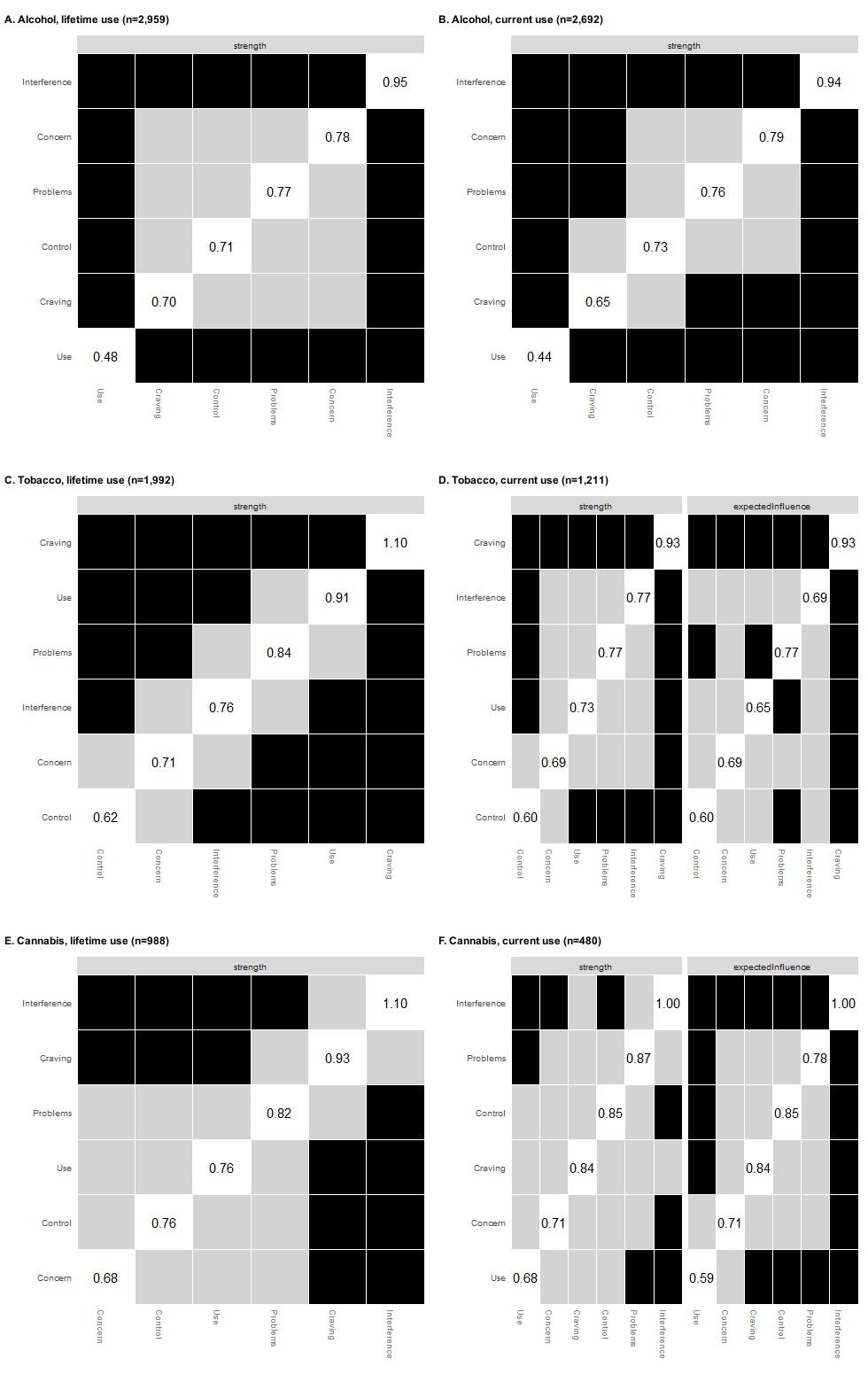


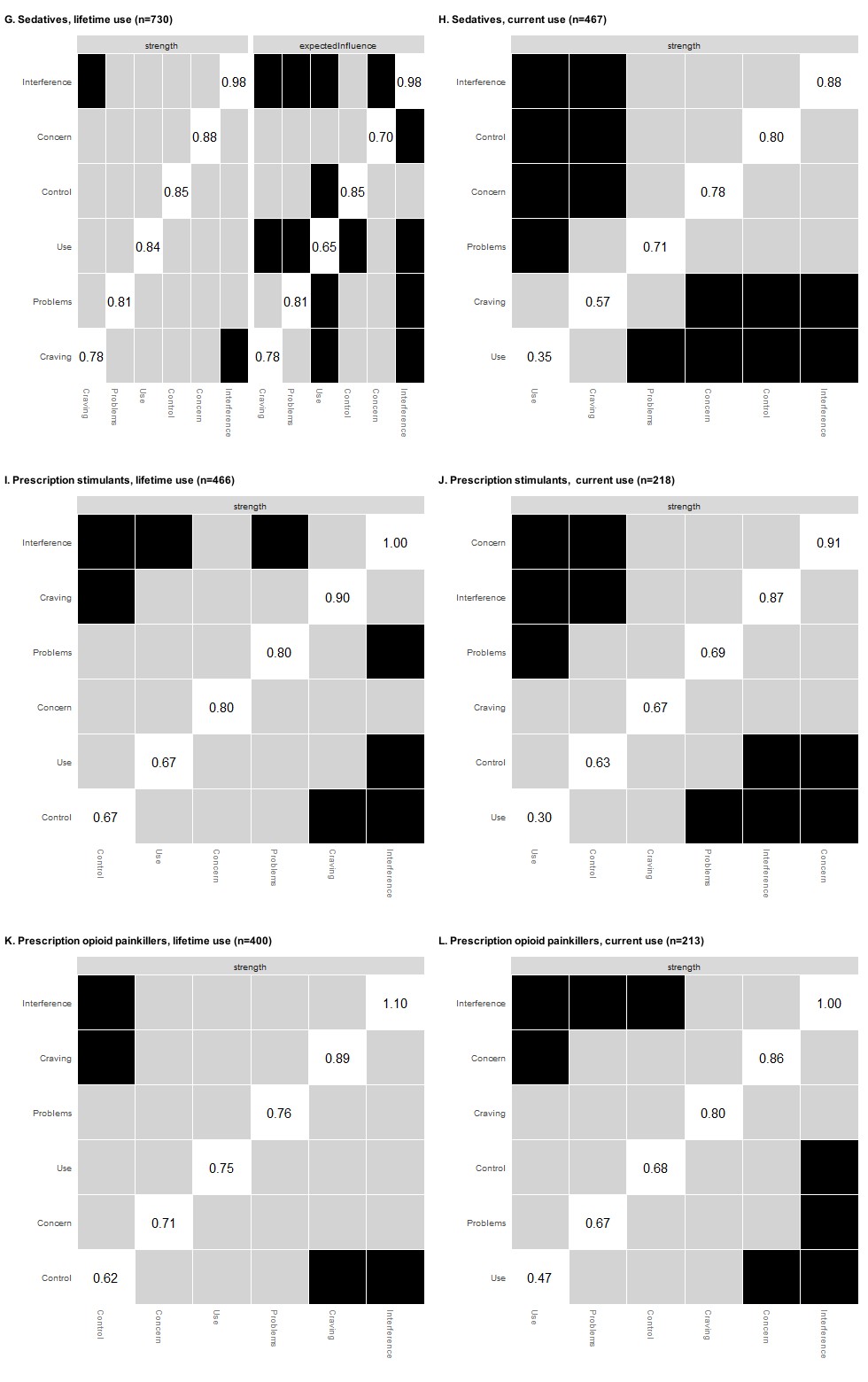


Centrality measures for each symptom are shown on the diagonal; black rectangles indicate centrality measures that differ significantly from each other (p<.05). Generally, strength is shown because expected influence was the same; where they differed, both are shown.

**Supplementary Figure 6: Centrality measures, substance specific networks, among current users**

Note: Alc = Alcohol; Tob = Tobacco; Can = Cannabis; Sed = Prescription sedatives; Stim = Prescription stimulants; Pain = Prescription opioid painkillers

**Note:** Each series represents both strength and expected influence centrality as they were the same, except for tobacco and cannabis.

**Supplementary Table 8. Edge weights that differ significantly between substances**

|  | **ALCOHOL and** | | | | **TOBACCO and** | | | |
| --- | --- | --- | --- | --- | --- | --- | --- | --- |
| Edges | Tobacco | Cannabis | Prescription stimulants | Prescription opioids | Cannabis | Sedatives | Prescription stimulants | Prescription opioids |
| Use-Craving | c | c |  | c | c | c | c | c |
| Use-Problems |  |  |  | c |  |  |  | c |
| Use-Interference |  | c | c | c | c | b | c | c |
| Use-Concern | a |  | c | c | a | c | c | c |
| Use-Control |  |  |  | a |  | b |  |  |
| Craving- Problems |  | b | a | a |  | a | c |  |
| Craving-Interference | c |  | b | a |  |  |  |  |
| Craving-Concern | b | a |  | c |  |  |  |  |
| Craving-Control | c | c | c | c |  |  |  |  |
| Problems -Interference | a |  |  |  |  |  | a |  |
| Problems-Concern |  |  |  |  |  |  |  |  |
| Problems -Control |  |  |  |  |  |  |  |  |
| Interference-Concern | b |  | a | a |  | a | c | c |
| Interference-Control | a |  |  |  |  | a |  |  |
| Concern-Control |  |  |  | a |  |  |  | a |

Results are shown for substance pairs that showed significant network variance, i.e., at least one edge-weight differed in the omnibus test (Table 2). Edge weights that differ are shown in grey, with the following p-values: a<0.05; b<=.01; c<=.001
